# Proposal of a Wearable Multimodal Sensing-Based Serious Games Approach for Hand Movement Training After Stroke

**DOI:** 10.3389/fphys.2022.811950

**Published:** 2022-06-03

**Authors:** Xinyu Song, Shirdi Shankara van de Ven, Shugeng Chen, Peiqi Kang, Qinghua Gao, Jie Jia, Peter B. Shull

**Affiliations:** ^1^ The State Key Laboratory of Mechanical System and Vibration, Shanghai Jiao Tong University, Shanghai, China; ^2^ The Department of Rehabilitation Medicine, The National Clinical Research Center for Aging and Medicine, Huashan Hospital, Fudan University, Shanghai, China

**Keywords:** stroke, EMG, FMG, IMU, movement training

## Abstract

Stroke often leads to hand motor dysfunction, and effective rehabilitation requires keeping patients engaged and motivated. Among the existing automated rehabilitation approaches, data glove-based systems are not easy to wear for patients due to spasticity, and single sensor-based approaches generally provided prohibitively limited information. We thus propose a wearable multimodal serious games approach for hand movement training after stroke. A force myography (FMG), electromyography (EMG), and inertial measurement unit (IMU)-based multi-sensor fusion model was proposed for hand movement classification, which was worn on the user’s affected arm. Two movement recognition-based serious games were developed for hand movement and cognition training. Ten stroke patients with mild to moderate motor impairments (Brunnstrom Stage for Hand II-VI) performed experiments while playing interactive serious games requiring 12 activities-of-daily-living (ADLs) hand movements taken from the Fugl Meyer Assessment. Feasibility was evaluated by movement classification accuracy and qualitative patient questionnaires. The offline classification accuracy using combined FMG-EMG-IMU was 81.0% for the 12 movements, which was significantly higher than any single sensing modality; only EMG, only FMG, and only IMU were 69.6, 63.2, and 47.8%, respectively. Patients reported that they were more enthusiastic about hand movement training while playing the serious games as compared to conventional methods and strongly agreed that they subjectively felt that the proposed training could be beneficial for improving upper limb motor function. These results showed that multimodal-sensor fusion improved hand gesture classification accuracy for stroke patients and demonstrated the potential of this proposed approach to be used as upper limb movement training after stroke.

## Introduction

Stroke is one of the most common causes of severe and long-term disability, affecting 15 million people each year worldwide (World Health Organization, 2018). Up to 60% of stroke survivors suffer from upper extremity impairments (Van Der Lee et al., 1999). The functional improvement of upper extremities primarily depends on the hand function (Kwakkel and Kollen, 2007). However, in the recovery process from upper extremity hemiplegia, the restoration of distal motor function comes later and is more strenuous than the restoration of proximal motor function (Twitchell, 1951). Intensive, repetitive, goal-oriented, and feedback-oriented movement training is critical to restoring neural organization (Ada et al., 2006) and reducing hand motor function impairment (Ada et al., 2006). In addition, depression (Robinson and Benson, 1981) and cognitive dysfunction (Madureira et al., 2001) are also common symptoms after a stroke. Patients often suffer from a decreased mental state, accompanied by a decline in attention, execution, and memory (Jaillard et al., 2009). Thus, recovery of the mental state and cognitive function also play an essential role in recovery (Song et al., 2019).

For hospital-based rehabilitation of low hand motor function, exoskeleton gloves are commonly used for passive training (Vanoglio et al., 2017). Patients with moderate to high hand function typically perform goal-oriented and activities-of-daily-living (ADLs)-related movements repeatedly under the guidance of therapists, such as pinching a pen or using a spoon to hold beans. However, therapy-assisted training is often challenging because of high costs and the required medical resources needed. The most common home-based approach is based on plans prescribed by clinicians, however these have low compliance and a high dropout rate due to boredom and a lack of motivation (Cox et al., 2003). In addition, studies have shown intense goal-oriented training has little value unless the stroke patient is engaged and motivated (Winstein and Varghese, 2018).

Robot-assisted systems have also been proposed for more intensive stroke therapy (In et al., 2015). Although these systems can improve hand function, an unassisted system could be more effective (Lum et al., 2002) for patients with mild to moderate hand dysfunction. Unassisted rehabilitation systems can be classified into three categories: camera-based, tangible-interaction-objects-based, and wearable-sensor-based. Rehabilitation systems that use cameras for motion tracking (Mousavi Hondori et al., 2013), (Liu et al., 2017a) can be accurate, however, this method involves privacy issues (Alankus and Kelleher, 2015). Also, the environment cannot be too cluttered (Cheung et al., 2013), and other people should not appear in the camera’s view to avoid skeleton merging (LaBelle, 2011). Tangible-interaction-objects-based rehabilitation systems (Ozgur et al., 2018), (Delbressine et al., 2012) enable users to interact through manipulating tangible digital devices. These systems can be easy to use for the elderly and can reduce a learning times (Apted et al., 2006). However, these systems are generally limited to a single training mode and have poor scalability. Another approach is using a wearable-sensor-based system, such as those based on a data glove (Sun et al., 2018), (Zondervan et al., 2016), surface electromyography (sEMG) (Yang et al., 2018), force myography (FMG) (Sadarangani and Menon, 2017) or inertial measurement units (IMU) (Friedman et al., 2014). Data gloves commonly use flex sensors, accelerometers, and/or magnetic sensors (Rashid and Hasan, 2019). Although data gloves can detect finger movements precisely, it is difficult for stroke patients to wear gloves due to spasticity (Thibaut et al., 2013).

Electromyography sensors have been widely utilized for hand movement estimation (Jiang et al., 2018) and rehabilitation systems (Chen et al., 2017). However, classification accuracy for stroke patients is typically much lower than that of healthy individuals due of neural damage (Cesqui et al., 2013). The combination of EMG and IMUs has produced better training systems (Liu et al., 2017b), (Lu et al., 2014). Electromyography has the advantage of directly measuring comprehensive information from muscle activity, though signal quality can be affected by sweat (He et al., 2013). Also, EMG-based recognition strategies are usually hyposensitive to low-strength gestures. Instead of directly measuring the muscle activity, FMG measures the contact pressures profiles caused by tendon slide of the wrist. This mechanism (Jung et al., 2015), and FMG sensor-based wristbands have been used to detect the various hand gestures (Dementyev and Paradiso, 2014), (Zhu et al., 2018). Force myography is more sensitive to low-strength gestures (Cai et al., 2020), and also less susceptible to sweating. Therefore, multi-sensor fusion could be a promising method to improve EMG-based recognition accuracy for stroke patients. In addition, to optimize the engagement of patients (Witmer and Singer, 1998), serious games have been designed and utilized in rehabilitation systems (Mohammadzadeh et al., 2015), (Heins et al., 2017), (Nissler et al., 2019). The effectiveness of serious games has been shown to show better results than conventional approaches for upper limb motor function rehabilitation (Tăut et al., 2017).

To the best of our knowledge, this is the first paper to propose wearable FMG-EMG-IMU serious games hand movement training for stroke patients involving Activities of Daily Living hand movements. We aimed to test the feasibility of such an approach and hypothesized that multimodal sensing would demonstrate higher hand gesture recognition classification accuracy for stroke patients during directed ADL-related hand movements as compared to single modality sensing. We also hypothesized that the proposed sensing and algorithm configuration could detect stroke patient hand gestures during the cognitive and motor tasks required in the serious games. Finally, we hypothesized that stroke patients would qualitatively be more enthusiastic about movement training based on this approach as compared to conventional rehabilitation.

## Methods

### General Structure

**FIGURE 1:**
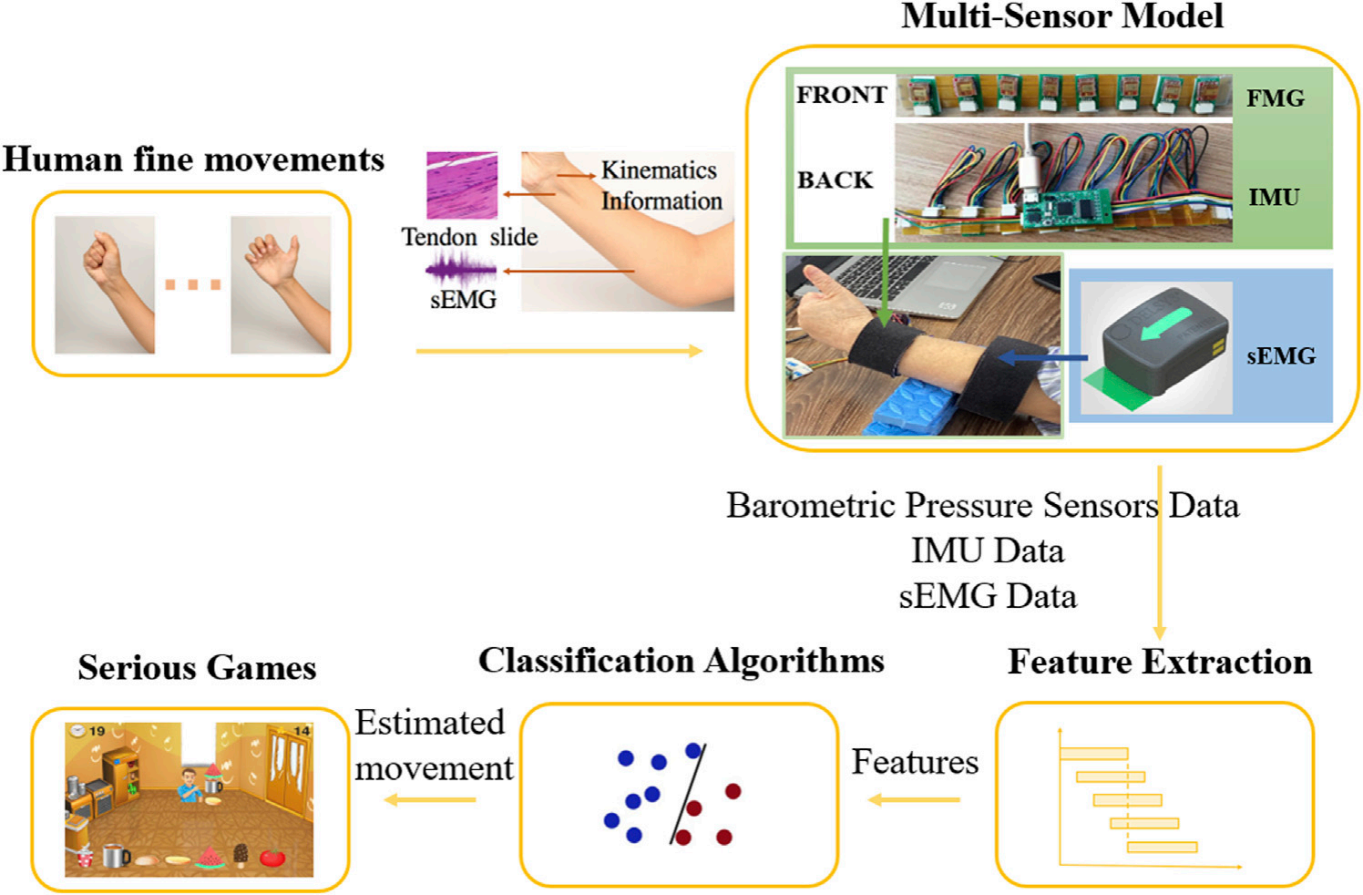
Wearable multimodal-serious game rehabilitation approach developed to improve upper extremity motor function and cognitive function after stroke. Patients performed 12 different ADLs-related fine movements. Kinematics data, morphology profile changes around the wrist and EMG data of forearm were extracted *via* IMU, FMG sensors and EMG sensors. Effective features were extracted from data after pre-processing and were put into classification algorithms. Two serious games were developed, and the predicted movement was used as input for the games, allowing the patients to interact with the targets in the game and get vision and sound feedback.

The general structure of the wearable multimodal-based movement training approach consists of five elements: human fine movements, multi-sensor model, feature extraction, classification algorithms, and serious games (Figure 1). Stroke patients first perform fine movements selected from the Fugl Meyer Assessment (FMA) (Fugl-Meyer et al., 1975). The physiological signal and kinematic signal of the user’s affected upper extremity are then collected by the multi-sensor model: the contact pressure profile is measured by barometric pressure sensors around the wrist, the EMG data is collected by wireless electrodes on the forearm, and kinematic data (including acceleration, angular velocity, magnetic field strength, and Euler angle) is collected by an IMU on the wrist. After preprocessing, features extracted from barometric sensor data, EMG data, and IMU data are put into the movement classification algorithms. Finally, the estimated fine movements are sent to the serious games. More details about this proposed approach are presented below.

### Upper Extremity Movement Selection

**FIGURE 2:**
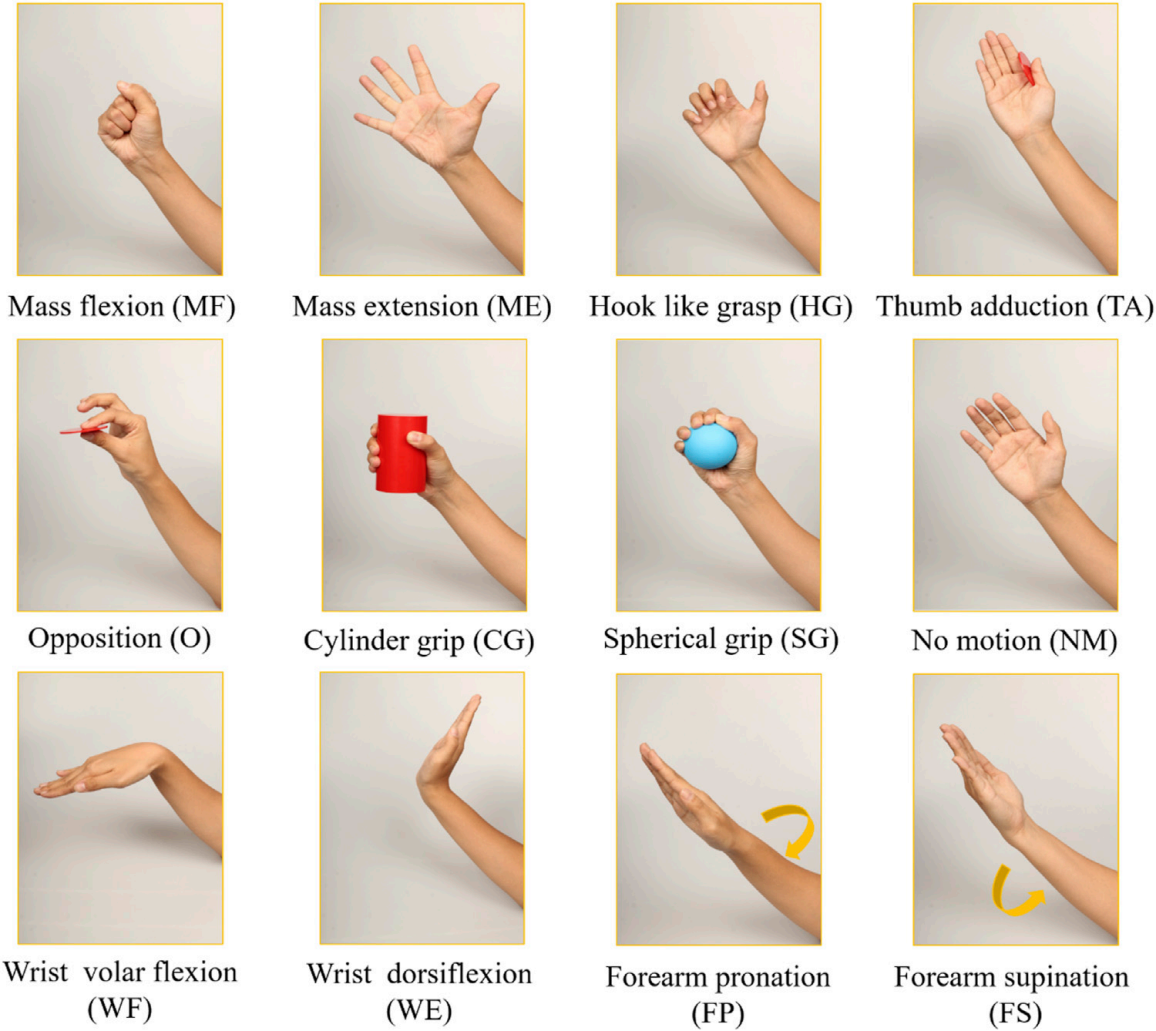
Hand gestures including 11 FMA movements (Fugl-Meyer et al., 1975) and one no-motion gesture.

The FMA is an effective and detailed evaluation tool for assessing motor function after stroke (Gladstone et al., 2002). It is the most widely used clinical assessment scale, and the test items are highly correlated to ADLs. Eleven upper extremity fine movements (Figure 2) are selected from the FMA and are suitable for motor function rehabilitation *via* a wearable multimodal-based system. The movements include hand movements: mass flexion (MF), mass extension (ME), hook-like grasp (HG), thumb adduction (TA), opposition (O), cylinder grip (CG), spherical grip (SG); wrist movements: wrist volar flexion (WF) and wrist dorsiflexion (WE); forearm movements: forearm pronation (FP) and forearm supination (FS). A no-motion (NM) movement is also included. Some selected movements, including HG, TA, O, CG, FP, and FS, are also included in another practical motor function scale - Wolf Motor Function Test (WMFT) (Wolf et al., 2001). These movements are highly related to ADLs, which are relevant for recovering stroke patients.

### Prototype Design

A wearable multi-sensor model for upper limb fine movement estimation was developed, containing six EMG sensors around the forearm and eight barometric pressure sensors plus one IMU around the wrist (Figure 1). There are multiple major superficial muscles around the forearm: the extensor carpi ulnaris, extensor digitorum, extensor carpi radialis longus and brevis, brachioradialis, pronator teres, flexor carpi radialis, flexor carpi ulnaris, and palmaris longus. Instead of applying a muscle-targeted layout, a low-density surface electrode layout was selected to detect the electromyographic signal of these muscles for practical use. Thus, six EMG wireless sensors from the Trigno Wireless EMG System (MAN-012-2-6, Delsys Inc., Natick, MA, United States) were selected and placed evenly around the forearm of the patient’s affected side, about 10 cm away from the elbow (Tchimino et al., 2021), covered and kept in place by an elastic band. The direction of sensors was parallel to the direction of muscle fiber. The placement of EMG electrodes and the number of electrodes used in this paper were informed by previous similar studies (Liu et al., 2014), (Yu et al., 2018).

During wrist and hand movements, tendons of the wrist shorten and lengthen, and muscles are deformed, resulting in large contour changes to the underside of the wrist. The optimal number of locations of the FMG sensors were selected based on previous recommendations in related research (Shull et al., 2019). Thus, a flex wristband containing eight barometric pressure sensors was developed to obtain contact pressure profiles around the wrist. Barometric sensors (MPL115A2, Freescale Semiconductor Inc., Austin, TX, United States) were covered by VytaFlex rubber and placed at the wrist near the distal end of the ulna to estimate the force myography of the tendon slide. The fourth and fifth pressure sensors of the eight sensors-flex-wristband were aligned to the center of the underside of the patient’s wrist, with other pressure sensors placed evenly on the inside and both sides of the wrist. A 9-axis IMU (BNO055; BOSCH Inc., Stuttgart, Baden-Württemberg, German) was mounted on the back of the flex wristband to detect kinematic information. The output data of the IMU included 3-dimensional accelerations, 3-dimensional angular velocities, 3-dimensional magnetic field strengths, and 3-dimensional Euler angles. FMG data and IMU data were transmitted to a microcontroller (STM32F401; STMicroelectronics N.V., Geneva, Switzerland) for processing and analysis.

Custom multi-threaded MATLAB (MathWorks, Natick, MA, United States) data collection software was developed to collect, synchronize, and process streaming sensor data. EMG data were collected at 1926 Hz, and FMG and IMU data were collected at 36 Hz. Also, a user-friendly instruction program (Supplementary Figure S1) was developed in MATLAB to instruct subjects to perform various movements during the training phase. Users were asked to perform movements corresponding to the text and pictures shown on the software interface for training data collection. The start button was used to start each trial of the test, and then the software would automatically time the current movement and change to the next movement. The stop button was used to stop the system. Data collection and instruction software were communicated by a virtual serial port. While each picture was shown (Supplementary Figure S1), corresponding triggers were transmitted to the data collection software *via* the virtual serial port in real-time. At the end of data collection, all data for each user was automatically saved.

### Testing Protocol

**FIGURE 3:**
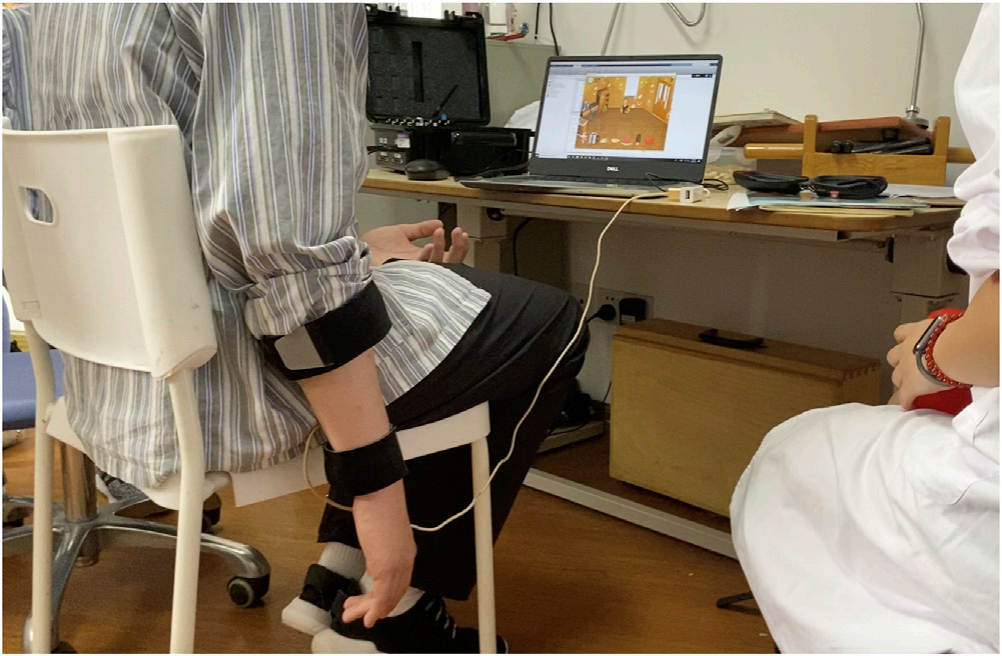
Example stroke patient playing a serious game while wearing the wearable multimodal-based system.

A clinical experiment was conducted to validate the estimation accuracy and practicality of the proposed approach (Figure 3). Ten stroke patients (Brunnstrom stage for Hand II-VI) (Table 1) were recruited in this experiment (Supplementary File S1 Patient inclusion criteria). Power analysis sample size calculation (power: 90%, alpha: 5%) was performed based on a cohort of healthy subjects from a previous related study (Jiang et al., 2020) to determine that 10 subjects were sufficient to detect differences in performance between multimodal and single sensor configurations. An experienced clinician was recruited to assist in conducting the experiment with all patients and record the special circumstances. The experiment was conducted in the Rehabilitation Medicine Department of Huashan Hospital (Shanghai, China). All the participants provided informed consent. The experiment was pre-approved by the Huashan Hospital Institutional Review Board (CHiCTR1800017568) and was performed in accordance with the Declaration of Helsinki.

**TABLE 1:** Stroke participant characteristics.

Sex (M/F)	8/2
Age (mean ± SD)	58.3 ± 18.09
Diagnosis (ischemic/hemorrhagic)	7/3
Hemiplegic side (left/right)	4/6
MMSE^a^ (mean ± SD)	27.7 ± 1.25
Brunnstrom stage for hand^b^ (mean ± SD)	4.5 ± 1.58
FMA upper extremity score^c^ (mean ± SD)	44.2 ± 13.9

Higher scores in MMSE, Brunnstrom stage, and FMA indicate better cognitive/motor function.

a Mini–Mental State Examination, used to measure the cognitive impairment, ranging from 0 to 30.

b Brunnstrom stages range from 1 to 6.

c Includes 33 FMA test items for upper extremity. Score range is 0–66.

An experienced clinician first explained the experimental process to each patient and told the patient to stop and report any discomfort during the experiment. The patient was then asked to sit on a chair without an armrest, so that the affected upper extremity naturally hung to the side of the body. Patients donned the device with the clinician’s assistance.

The experiment was divided into two phases: the training phase and the game phase. At the start of the training phase, the clinician explained all the movements to the patients in detail and showed instructional pictures to them. Next, patients were asked to perform movements following the instruction software we developed (Supplementary File S2 Interface of the instructional software) to get familiar with the movements and the system. The software shows the text and pictures of the current movement and the movement that comes next. Then, patients were asked to perform five formal trials in the training phase, with 1-min breaks in between. Each trial consisted of the data collection of 12 movements, and each movement lasted 6 s, with a 4-s break between movements.

After finishing the training, patients rested for 10 min while watching a game demo video to get familiar with two serious games. Then, patients started to play two movement-estimation-based serious games. Each game session consisted of five trials. Six patients finished all the 10 trials, two subjects lack of one trial, and two subjects quit due to back and waist fatigue when two trials were left. After completing the serious games, patients were asked to fill out a questionnaire (Table 2) about their experience of using this serious-games rehabilitation system. There were 10 questions, each of which could be answered with “strongly agree,” “agree,” “neutral,” “disagree,” and “strongly disagree.” Besides, we also solicited opinions from patients on improving this system.

**TABLE 2:** Questionnaire for Serious-Games Rehabilitation system.

Symbol	Questions
1	Does the game make you more enthusiastic about rehabilitation?
2	Were you relaxed and happy while playing the games?
3	Did you feel frustrated while playing the games?
4	Are the serious games challenging?
5	Was your body uncomfortable while playing the game?
6	Was the training part before the games a burden to you?
7	Do you think the game is suitable for home-based rehabilitation?
8	Is this sleeved sensor setup more practical than a glove setup?
9	Do you think the game is beneficial for improving your cognitive function?
10	Do you think the game is beneficial for improving your upper limb motor function?

### Serious Games Design

**FIGURE 4:**
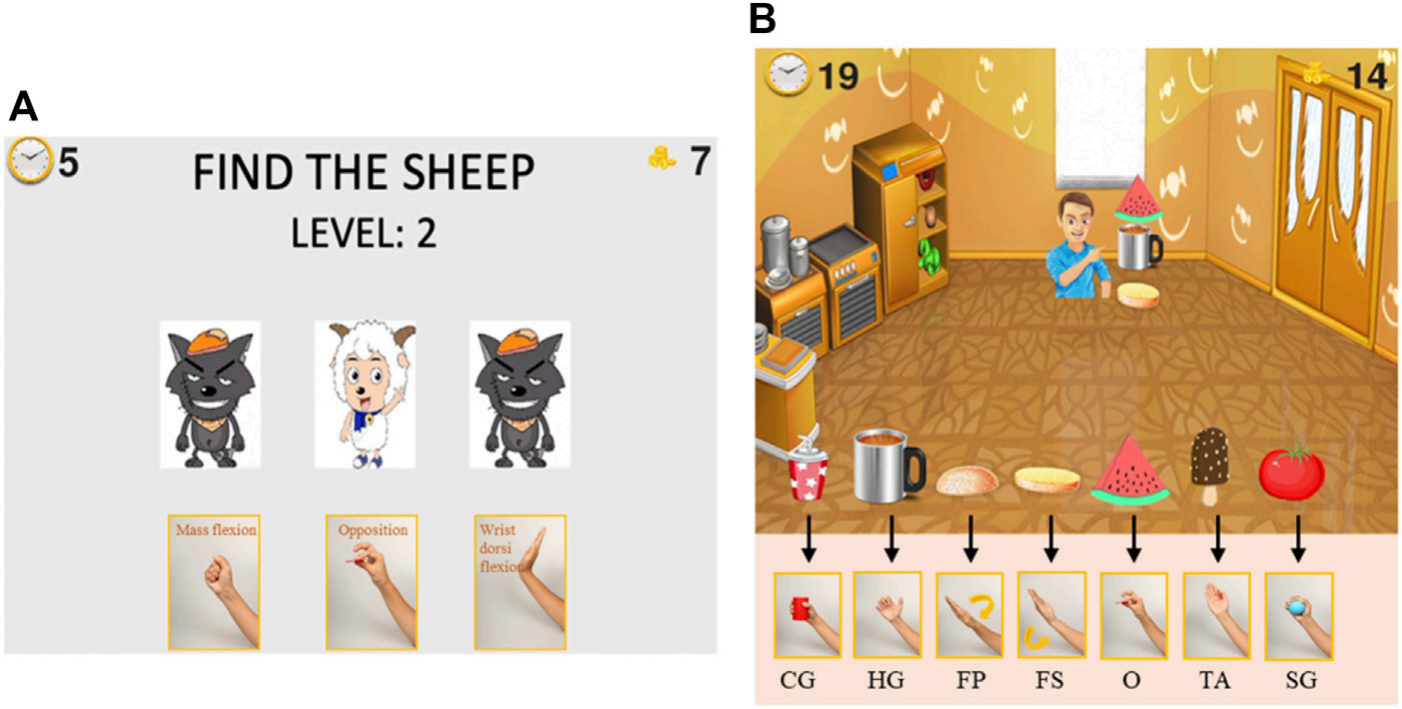
Serious games for upper extremity motor function and cognitive function rehabilitation. Patients select the correct target in the serious game and perform the corresponding movement with their affected hand. **(A)**, *“Find the Sheep”* game: find the location of the sheep card at the end of each round and perform corresponding movements. **(B)**, *“Best Salesman”* game: perform corresponding movement to provide customers with the food they need. The corresponding movements are only shown during training to stimulate cognitive rehabilitation.

Two serious games (Figure 4) were developed based on movement estimation. The games provide visual and audio feedback to the patients. When patients perform each correct movement, the text “excellent” appeared on the screen, and the score on the screen increased by one. Patients also heard a positive audio cue. The games were written in python based on the pygame library.

The game *“Find the Sheep”* was designed for both motor and cognitive function training. Patients need to concentrate during the whole game and perform the required movements. Three cards appeared in the game interface, with a sheep and two wolves on the front. Then, all three cards were flipped over such that the animals were hidden and randomly swapped positions. After swapping, the patient was required to find which card is the one with the sheep and perform the corresponding hand movement shown below that card.

The score in this serious game represents the number of times participants successfully located the sheep card and made the corresponding gesture. The 12 movements are divided into four groups for *“Find the Sheep,”* displayed in different game rounds (Supplementary File S3 Grouping of the different movements). Many of the movements we selected are similar, such as the spherical grasp and the cylinder grasp. By dividing the movements into several groups, the real-time recognition accuracy of the system is improved. The system loads the classification model trained for the current movement group during the game. The game has multiple difficulty levels. The higher the difficulty level, the more times the cards will be rearranged.

The game *“Best Salesman”* was designed to train motor function and improve performance in ADLs. In this game, the user owns a grocery store that sells seven types of food. Customers keep coming to the store to buy one to three types of food. Users need to pass the right food to the customers by performing the correct corresponding movement: hold a cup, take a cup with a handle, cover top burger bread, hold bottom burger bread, pinch a piece of watermelon, lateral pinch a popsicle, and hold a tomato. Patients can intuitively know which hand gestures should be performed when they see the object pictures, like in normal daily activities. The score in this serious game represents the number of objects that participants successfully “sold” to customers. Like the *“Find the Sheep”* game, different movements are divided into groups to increase the accuracy of the classification model for *“Best Salesman”* (Supplementary File S3 Grouping of the different movements).

### Signal Processing

Custom multi-threaded MATLAB (MathWorks) data collection software was developed, in which triggers were added to different sensor data in real-time for data synchronization. Also, Data of different movements were segmented automatically based on triggers in the data collection code, which correspond to different movements. During the transition period between movements, related muscle activities erupt and cause a larger EMG amplitude. Besides, stroke patients are generally older and slower to respond. Thus, for the data collected in the training phase, the first 2 s and the last 0.5 s of each movement are removed to reduce interference.

EMG data were collected at 1926 Hz, and FMG and IMU data were collected at 36 Hz. For EMG segmentation, overlapped segmentation with a window length of 200 ms and an increment of 50 ms has a short response time while ensuring accuracy, which is suitable for the real-time movement classification (Oskoei and Huosheng Hu, 2008). Considering the performance of the algorithm and the synchronization of EMG, FMG, and IMU data, overlapped segmentation with a window length of 222 ms and a step size of 55.6 ms was adapted to divide the raw EMG data into windows. Disjoint segmentation with a window size of 55.6 ms was used to segment both the FMG and IMU data. The FMG data that exceeded the measuring range was deleted during the preprocessing phase.

Time-domain features are very effective in EMG pattern recognition (Rechy-ramirez and Hu, 2011). Four reliable time-domain features (Supplementary File S4 Feature formulas) were selected and extracted from EMG signals: Mean Absolute Value (MAV), Waveform Length (WL), Zero Crossings (ZC), Slope Sign Changes (SSC) (Englehart and Hudgins, 2003). MAV contains information about a signal’s strength and amplitude. WL reflects the signal’s complexity. ZC and SSC reflect the frequency information of the signal, both containing a threshold ( ϵ=4e−6 ) to reduce noise interference. A frequency-domain feature was also selected from the EMG signal: Auto-Regressive Coefficients (AR), which describes each signal sample as a linear combination of previous samples plus white noise error terms $e_{k}$ (Proakis and Monolakis, 1996). The fourth-order AR was used (Supplementary File S4 Feature formulas). Also, MAV was chosen as the feature of FMG and IMU signals.

In total, six channels of EMG data, eight channels of FMG data, and 12 channels of IMU data were used. Eight features were extracted from each window of each EMG channel. Meanwhile, the MAV of each window of each FMG and IMU channel was calculated. Thus, 48 EMG features, eight FMG features, and 12 IMU features result in a 68-dimensional feature array. Each channel’s data was scaled and normalized *via* zero-mean normalization by using the mean value and standard deviation from each respective trial.

Signal processing for real-time classification in serious games was the same as signal processing during the model training, features were extracted from real-time data of sensors on patients in MATLAB, after which they were transferred to the game in real-time *via* TCP/IP communication. Then, the trained models were loaded and used to classify movements. Finally, the estimated movement was used as input to the game, allowing patients to choose targets by performing the corresponding movement. The users were given 1 s to react and perform the target movements in each round of the serious games. Correct selections were determined by classification majority voting of a 10 frame window 1 s after the cards stopped moving in the *“Find the Sheep”* game and 1 s after the new image was shown in the *“Best Salesman”* game.

### Algorithm

This system uses online linear discriminant analysis (LDA) for real-time classification, because it can simplify the computational complexity, shorten the time, and still produce accurate results (Englehart and Hudgins, 2003), and is robust (Kaufmann et al., 2010). The LDA classification algorithm is based on Bayes decision theory and the Gaussian assumption. The discriminant function is defined as:

$$\delta_{k}\left(x\right)=x^{T}\Sigma^{-1}\mu -\frac{1}{2}\mu_{k}^{T}\Sigma^{-1}\mu_{k}+\log\pi_{k}\;$$

Where x is the input vector, Σ is the covariance matrix, $\mu_{k}$ is the k class’s mean and $\pi_{k}$ is the prior probability of class k .

In previous research, decision tree (DT) (Ting Zhang et al., 2013), k-nearest neighbor (KNN) (Zhang et al., 2015), random forest (RF) (Bochniewicz et al., 2017), and support vector machine (SVM) (Cai et al., 2019) have also been used for stroke rehabilitation classification. Apart from LDA, these four algorithms were also tested for movement classification in the study. Entropy was used to evaluate the quality of split in DT. Euclidean distance was used as the distance metric, and the number of neighbors was set to three to inspect in the KNN model. In the RF model, the number of trees and the minimum samples of leaf were determined as 40 and 1. The linear kernel was used in SVM, and the penalty parameter *C* was determined as 100.

### Model Training and Evaluation

To validate the efficiency and accuracy of the proposed multimodal-based hand gesture classification on stroke patients, the average accuracy in classifying the 12 directed hand movements was calculated. Five trials in the training phase were used to perform an offline test, using leave-one-out cross-validation. Training data and test data for offline testing were both taken from the 2 to 5.5 s. A confusion matrix was created to display the recognition rate of each gesture and the misclassification between gestures.

To determine the performance of different sensor configurations, the accuracy of single, double, and triple sensor-based classification algorithms were calculated separately. Also, the confusion matrixes of EMG-alone-based hand gesture classification and FMG-alone-based hand gesture classification were created to show the contribution of different sensors on different gestures. Also, the roles of FMG and EMG in multimodal hand gesture recognition were analyzed, mainly for fine finger movements which are easily misclassified from each other, such as CG and SG, and MF and CG. When two pairs of hand gestures were classified: CG and SG, and MF and CG, the most contributed features among all 56 FMG and EMG features were selected. Gini importance of the Random Forest was used for computing feature importance (Ling-ling et al., 2015). Feature importance was normalized by criterion reduction, and the most essential 10 features were sorted to demonstrate how these sensing modalities compensate for each other. In addition, Pearson correlation coefficients (PCCs) between EMG-based offline accuracies, FMG-based offline accuracies, and FMG-EMG-IMU-based offline accuracies for all subjects were calculated to study the correlation between the performances of different physiological information-based movement recognition. The performance of DT, KNN, RF, and SVM was also analyzed as compared with LDA.

To further explore the characteristics and potential uses of different sensing modalities in stroke rehabilitation, the correlation between subjects’ upper limb motor function and their different information-based hand gesture classification accuracies was analyzed. PCCs between the Fugl Meyer Upper Extremity (FMUE) scores of stroke patients and their offline accuracies of EMG-based, FMG-based, and FMG-EMG-IMU-based hand gesture classification were calculated, respectively.

Because it was difficult to tell whether the system classified incorrectly or the patient did not successfully perform the right movement during the serious games. The performance of real-time classification after grouping (Supplementary File S3 Grouping of the different movements) was simulated and validated. We applied cross-validation on five trials for each subject to validate the real-time performance. Four trials were used as training data, all of which ranged from the 2nd to the 5.5th second. The first 10 samples starting from the first second of the leftover trial were used to test the model. Also, different cutoffs of training and test data were analyzed (Supplementary File S5 Different cutoffs - Statistical analysis), and the classification accuracies of each group (Supplementary Table S1) were calculated with optimal cutoff settings.

In addition, the results of the questionnaires were analyzed to define the patients’ subjective feelings about using the proposed rehabilitation approach. Patients’ suggestions were also examined and served as important references for future improvements to the proposed approach. The performances of stroke patients playing serious games were also studied. The average scores of all the subjects for each trial in the serious games “*Find the Sheep*” and “*Best Salesman*” were calculated and analyzed.

### Statistical Analysis

IBM SPSS Statistics Version 26 was used for statistical analysis. Shapiro–Wilk normality test was used to confirm data were normally distributed (*p* > 0.05). One-way repeated analysis of variance (ANOVA) was conducted to assess if there were differences between using different sensor configurations and different algorithms. If there was a difference, LSD procedure was used for *post hoc* analysis. PCC was used to assess the correlation between patients’ motor function and gesture recognition accuracy, the correlation between different sensor configuration-based classifications, and the correlation between subjects’ average scores in two serious games. The statistical significance was set to *p* < 0.05.

## Results

**FIGURE 5:**
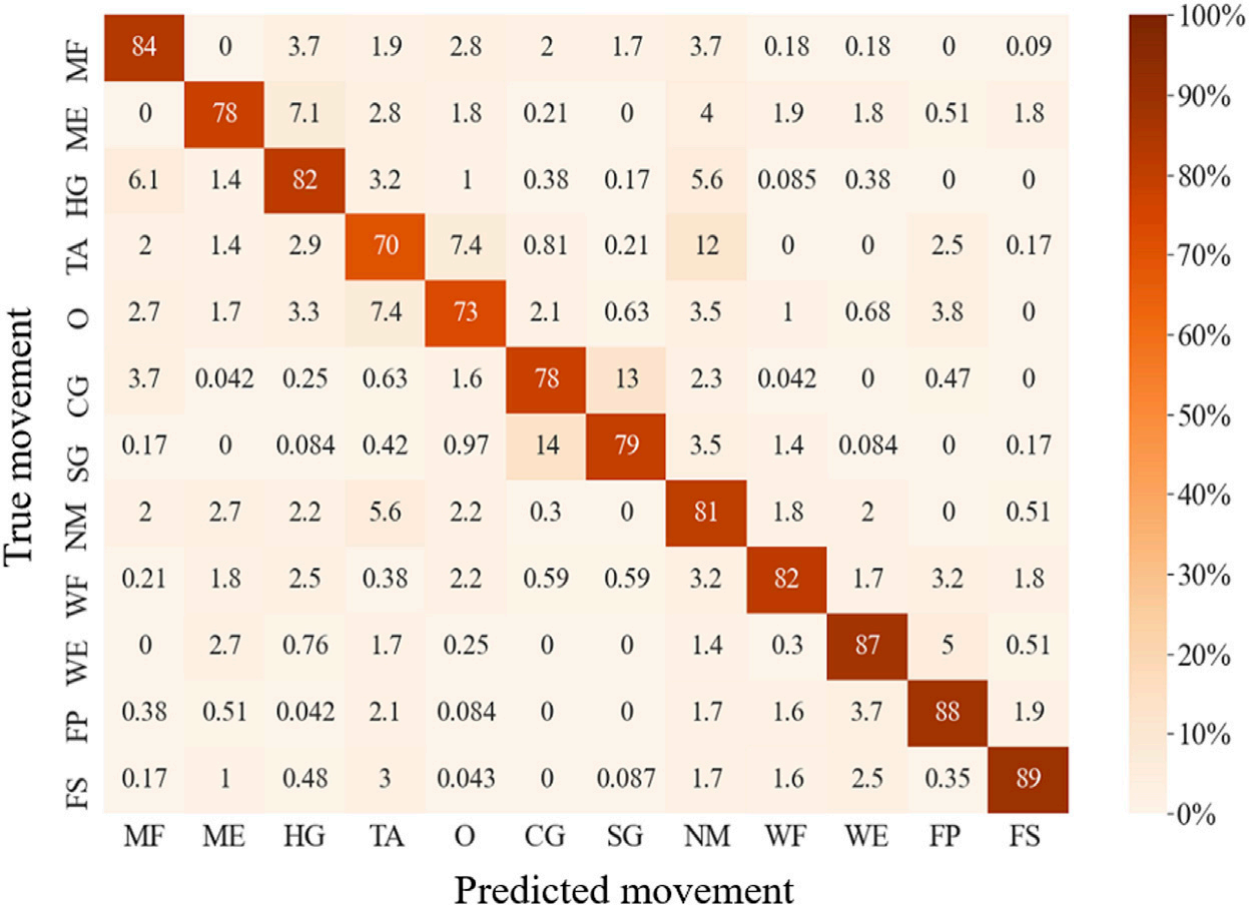
Confusion matrix for wearable multi-sensor-based movement offline classification.

**FIGURE 6:**
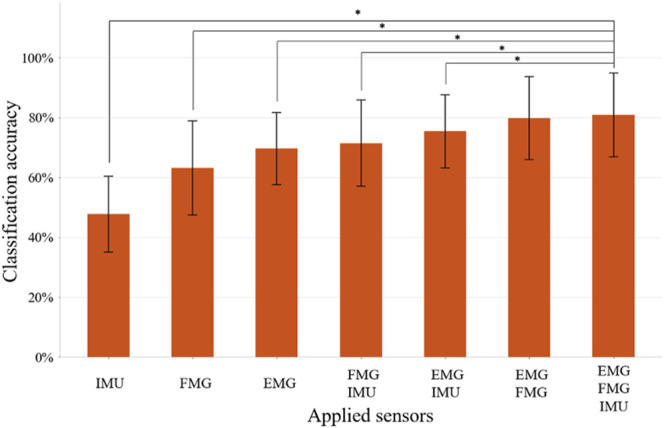
The classification accuracy of 12 movements using different combinations of sensors. Bars represents one SD. * represents statistical significance (*p* < 0.05). The application of three sensors significantly improves the recognition accuracy compared to the application of IMU, FMG, EMG, FMG+IMU, or EMG+IMU, respectively.

The LDA-based offline classification accuracy of 12 movements for each subject ranged from 64.3 to 96.3%, with an average accuracy of 81.0% for all 10 patients. The predicted accuracies of each movement range from 70.5% (for TA) to 89.1% (for FP) (Figure 5). CG and SG were most likely to be misclassified with each other. In addition, TA and O were commonly misclassified, and TA was often misclassified as NM. ME was often mistakenly recognized as HG, and HG was often misrecognized as MF or NM. Also, WE and FP were sometimes misclassified. The classification accuracy of using IMU alone, FMG alone, EMG alone, FMG and IMU, EMG and IMU, EMG, and FMG or all the sensors were 47.8, 63.2, 69.6, 71.5, 75.4, 79.7, and 81.0%, respectively (Figure 6). There is a significant improvement using FMG, EMG, and IMU together, as compared with: IMU alone, FMG alone, alone, EMG and IMU together, or FMG and IMU together. In addition, there was no significant difference between using FMG-EMG-IMU model and using FMG-EMG model.

**FIGURE 7:**
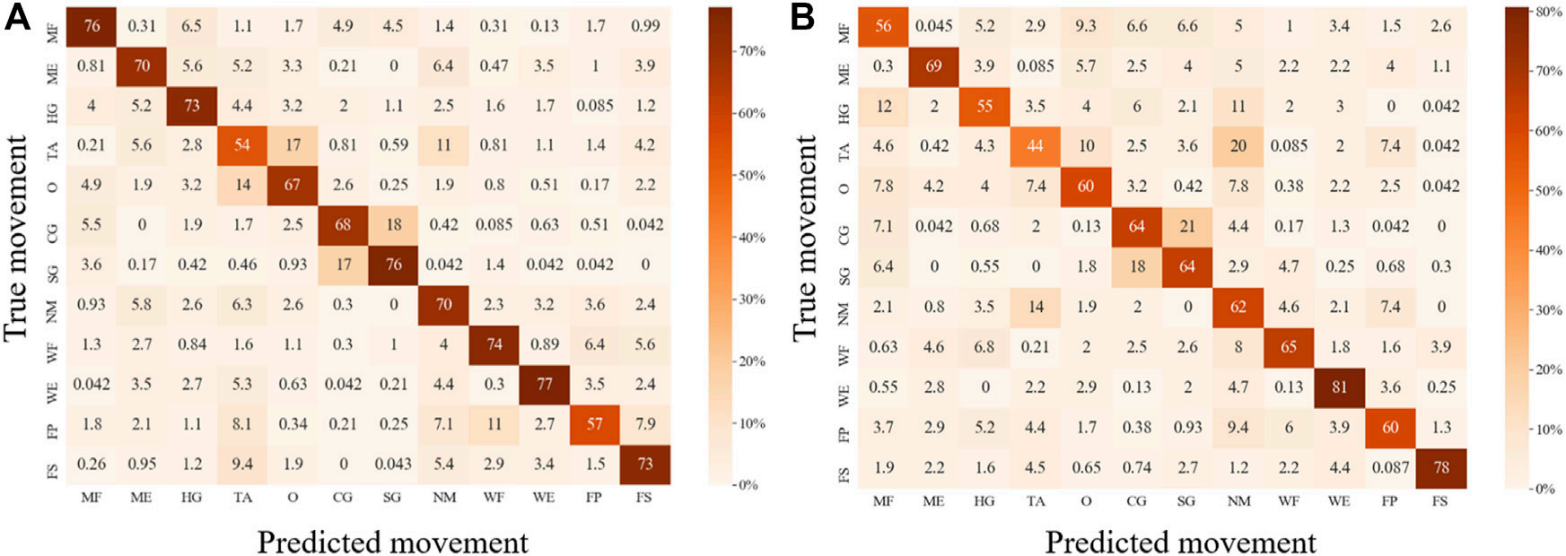
Confusion matrixes for movement classification based on different sensor configurations. **(A)**, Confusion matrix for EMG-based movement classification. **(B)**, Confusion matrix for FMG-based movement classification.

**FIGURE 8:**
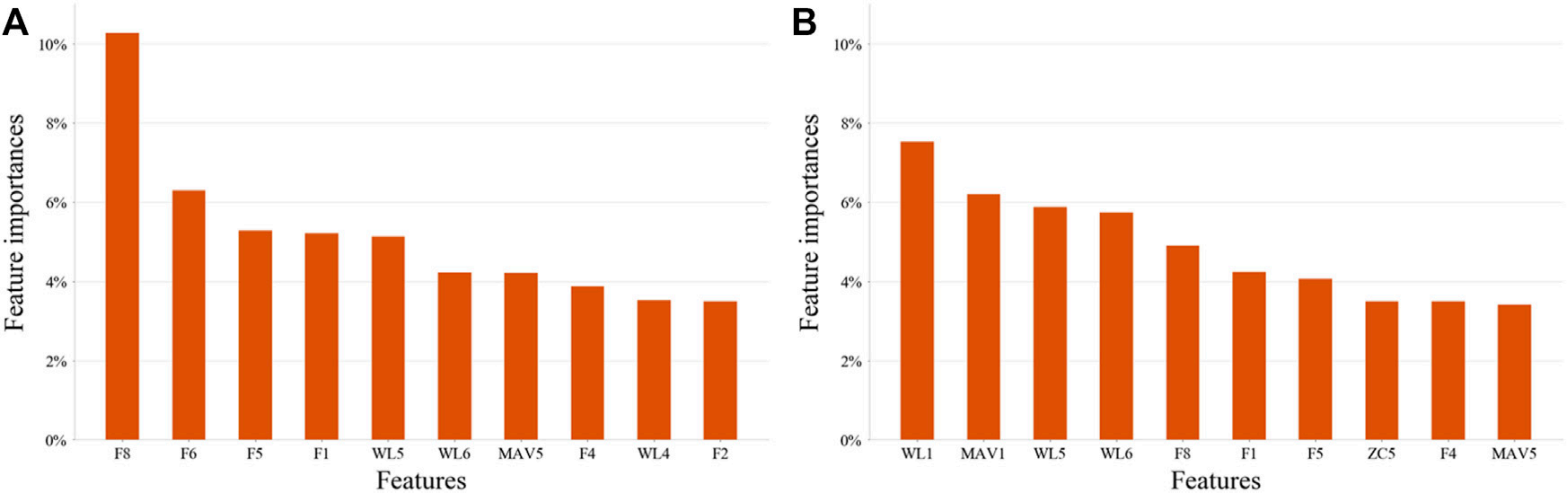
The most important 10 features on two pairs hand gestures recognition by using EMG-and-FMG-model. **(A)**, Ten features contributed most to the classification of hand gesture SG and CG. **(B)**, Ten features contributed most to the classification of hand gesture MF and HG.

For both EMG-based and FMG-based hand gesture classification (Figure 7), recognition performances on some gestures were the same. For example, CG and SG were easily misclassified with each other in both models. However, when recognizing other gestures, different models performed differently. For instance, MF was often mistakenly identified as HG when the EMG-based model was applied, and MF was easily misrecognized as O when the FMG-based model was used. However, when two sensing modalities EMG and FMG were used together, the misclassify rate of CG and SG was decreased. The 10 features that contributed the most were six FMG-related features and four EMG-related features (Figure 8). Similarly, six EMG-related features and four FMG-related features contributed most to classify MF and HG (Figure 8). There’s a correlation between classification accuracies of the EMG-based model and classification accuracies of the FMG-based model (*r* = 0.69, *p* < 0.05), and there’s also a correlation between classification accuracies of the EMG-based model and classification accuracies of the FMG-EMG-IMU-based model (*r* = 0.73, *p* < 0.05). There’s a high correlation (*r* = 0.94) between classification accuracies of the FMG-based model and classification accuracies of the FMG-EMG-IMU-based model (*p* < 0.05). The average offline classification accuracies of applying DT, KNN, RF, and SVM were 62.7, 72.9, 78.4, and 80.9%, which were lower than LDA’s 81.0% accuracy. However, LDA only had significant difference with DT and KNN (*p* < 0.05).

**FIGURE 9:**
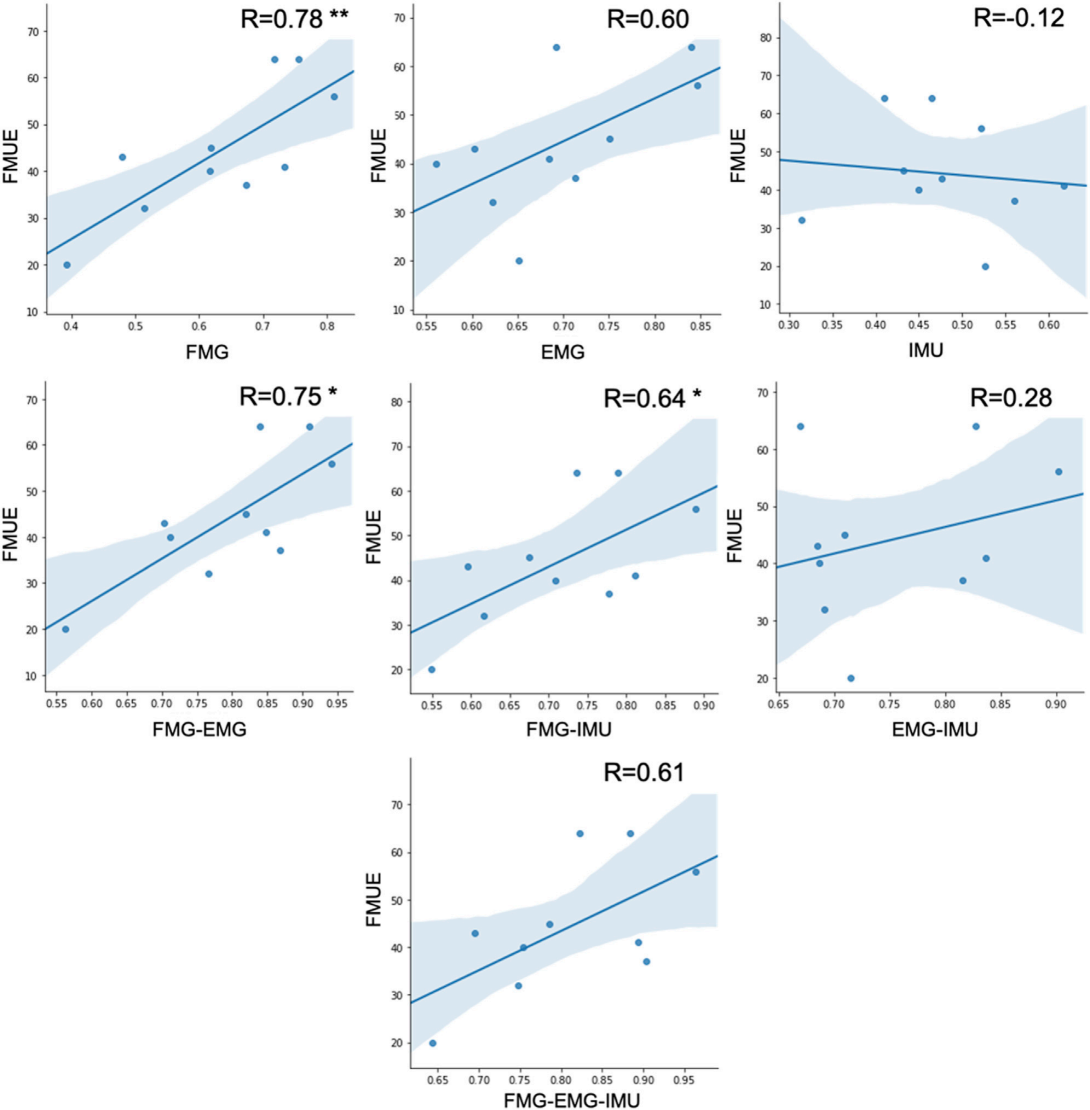
The correlation between FMUE scores and different sensing modality-based classification accuracies for all subjects (* represents *p* < 0.05, ** represents *p* < 0.01).

There is a significant correlation between FMUE and offline classification accuracies of the FMG-based model (*r* = 0.78, *p* < 0.01). There is also a significant correlation between FMUE and offline classification accuracies of FMG-EMG-based model (*r* = 0.75, *p* < 0.05). Fugl-Meyer upper extremity and offline classification accuracies of FMG-IMU-based model are significantly correlated (*r* = 0.64, *p* < 0.05). However, there’s no significant correlation between FMUE and the offline classification accuracies of the EMG-based model (*r* = 0.61, *p* = 0.065), or the IMU-based model (*r* = −0.12, *p* = 0.748), or the EMG-IMU-based model (*r* = 0.28, *p* = 0.438), or the FMG-EMG-IMU-based model (*r* = 0.61, *p* = 0.063), respectively (Figure 9).

By splitting up the movements into groups, the accuracies of simulation real-time classification ranged from 80.7 to 84.7% for each movement group, and the simulated classification accuracies were 82.5 and 83.6% for the groups used in the *“Find the Sheep”* game and the *“Best Salesman”* game, respectively. Different combinations of cutoffs were applied, and the simulated real-time classification accuracies of two serious games were over 90% when using ideal cutoffs (Supplementary File S6 Different cutoffs - Results).

**FIGURE 10:**
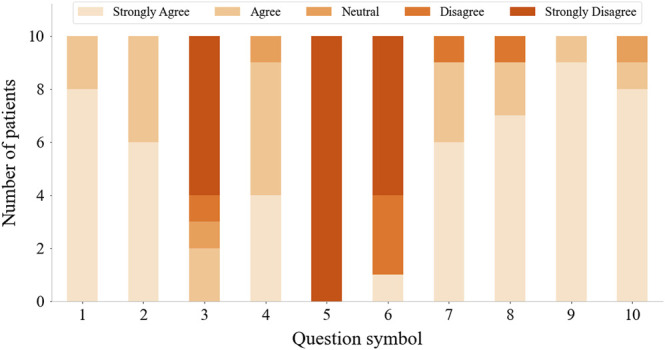
Questionnaire results from questions in Table 2.

**FIGURE 11:**
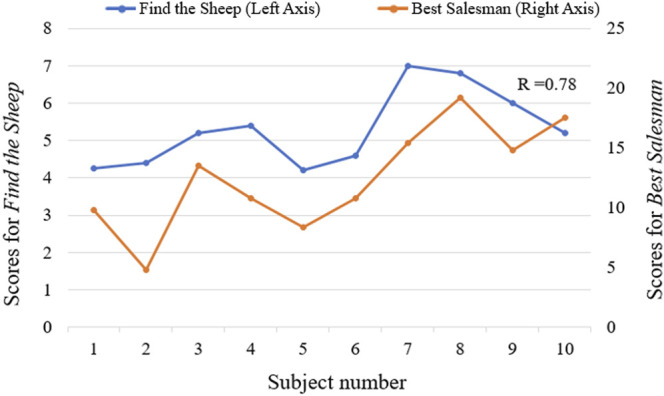
The correlation between average scores of serious games *“Find the Sheep”* and *“Best Salesman”* (*r* = 0.78, *p* < 0.05).

All stroke patients who participated in the serious games filled out the questionnaires (Figure 10). The majority of patients strongly agreed that the serious games made them more enthusiastic about rehabilitation and that they felt relaxed and happy while playing the games. 60% of the patients didn’t feel frustrated during the games, while 20% felt a little frustrated. 60% of patients strongly agreed that the games were challenging. None of the patients experienced any upper limb discomfort during the games. 90% of patients strongly disagreed or disagreed that the training part before the games was a burden. 90% of patients strongly agreed or agreed that the proposed system is suitable for home-based rehabilitation and is more practical than wearing a glove. Most patients also strongly agreed that the proposed training is beneficial for improving both upper limb motor function and cognitive function. The patients expressed other thoughts and suggestions about the proposed system. Three patients thought the proposed system was beneficial for brain and neurological restoration. Two patients mentioned that the proposed training was entertaining, which increased the attractiveness of rehabilitation. Also, two patients thought the selected movements were significant, involving ADLs functional training such as grasping a cup. One patient expressed that the training strengthened his confidence. The patient with the lowest classification accuracy indicated that the sensing part needed improvement and the game time should be shortened. Two patients suggested the games should be more challenging, while two other patients thought it was too hard for them to complete the right movement within the prescribed time and that the games should be simpler and slower in the future. The average scores across all the subjects for each trial in the serious game *“Find the Sheep”* were 5.6, 4.9, 5.8, 5, and 5.3. In addition, the average scores of each trial in the serious game *“Best Salesman”* were 11, 13.5, 11.5, 11.4, and 13.4. Also, there’s a correlation (*r* = 0.78) between subjects’ average scores of playing *“Find the Sheep”* and patients’ average scores of playing *“Best Salesman”* (*p* < 0.05) (Figure 11).

## Discussion

We proposed a serious games movement training approach to recognize the movement of stroke patients’ affected sides *via* a multi-sensor fusion model and provide patients with serious games and feedback for upper limb fine movement and cognition training after stroke.

In previous research, most studies focused on pattern recognition for stroke rehabilitation systems (Yang et al., 2018), (Chen et al., 2017), but only healthy subjects were included, or the motion of each patient’s unaffected side was included to perform bilateral training (Leonardis et al., 2015), (Lipovsky and Ferreira, 2015). Few studies have worked on the affected-side-based motion recognition of stroke patients. Lee et al. (Lee et al., 2011) recruited 20 stroke patients with chronic hemiparesis and selected six functional movements. Ten surface electrodes were applied to record EMG signals. The mean accuracy of moderate-function patients and low-function patients was 71.3 and 37.9%, respectively. Zhang et al. (Ping Zhou and Zhou, 2012) applied high-density electrodes to classify 20 movements of 12 stroke patients, resulting in a classification accuracy of 96%. Castiblanco et al. (Castiblanco et al., 2020) proposed a study of hand motion recognition *via* EMG. Healthy subjects, stroke subjects without hand impairments, and stroke patients with impairments were included. In their research, hand gestures were separated into several groups, and each group contained two to five movements; in their study, the average recognition accuracy for stroke patients with impairments was 85%. The classification accuracy for 12 movements of the proposed system is 81.0%, which is lower than the system applying high-density EMG. However, we covered more movements related to ADLs, and obtained higher classification accuracy compared to other previous research.

Our results verify that the multi-sensor fusion method significantly improves accuracy over single sensor-based pattern recognition. The accuracy of applying both EMG and FMG was close to the accuracy of using all sensors. EMG and FMG contribute the most to movement recognition, which may be due to the IMU being placed on the wrist and most of the movements we selected being finger movements. The EMG-based model and FMG-based model showed different performances on gesture recognition. The information from these two models can be used to compensate for each other to increase the system’s robustness. In addition, there’s a significantly high correlation between subjects’ FMG-based hand gesture classification accuracies and their FMG-EMG-IMU-based hand gesture classification accuracies. It indicates FMG information has the most influence on the multi-sensor fusion model. The multi-sensor fusion model we proposed contains more information and improved robustness, improving the accuracy of motion recognition on the affected side and expanding the range of users.

The patients we recruited range from high motor function to low motor function. Results showed a significant correlation between subjects’ upper limb motor function and the offline accuracies of FMG-based hand gesture recognition. It indicates that wrist-tendon-slide-related information can be used to assess the upper limb motor function of stroke patients. Also, it’s interesting that subjects showed consistency in two different serious games. In addition, when subjects were playing the game, there was not an obvious improvement in their scores over time. We assumed that the main reason is games were difficult for the subjects. Most subjects were older people and suffered from brain injuries, so their learning curve may be relatively long. The serious games’ settings need to be further considered in future research.

Serious games were designed for cognition and upper limb fine movement training. Feedback is sensory information provided during or after task performance. Training should include appropriate feedback, which can help improve movement (Winstein et al., 1999). The proposed system provides extrinsic feedback, including visual feedback and audio feedback, which has been shown to make users more enthusiastic in training (Burke et al., 2009), (Ferreira and Menezes, 2020), (Hocine et al., 2015).

To improve the effectiveness of the system and the patient’s experience of playing serious games, we grouped 12 movements during the real-time movement classification (Supplementary File S3 Grouping of the different movements) based on experience and prior knowledge so that three movements that are not easily confused for each other were grouped together. According to the results presented by the confusion matrix, it was verified that our grouping situation was almost ideal, avoiding the situation where movements that are easily misclassified appear in the same group. Spherical grasp and cylinder grasp are similar and easily misclassified with each other. In addition, the thumb adduction and opposition are also very similar; especially compared to other movements, it is more difficult for patients to exert force correctly and effectively complete these two movements, which also makes these two movements not only easy to mix up with each other but also easy to misclassify as no motion.

Because of poor hand function in some patients, and some of the movements we choose having similarities, it is difficult to observe from the outside what movement the patient is performing in many cases. Therefore, in real-time classification games, when patient movement was determined to be incorrect, it was difficult to tell whether the system classified incorrectly or the patient did not complete the right movement. Therefore, we can not validate the real-time performance of the multi-sensor model in this experiment. We used training data to simulate real-time classification through cross-validation. This simulation process was the same as the process we used in the games. We found through this research that the cutoff settings of training data and testing we used in the experiment was not optimal: the simulated real-time accuracy is relatively low, and some patients reported that the games moved too fast to complete the movement. The response time and execution time of patients should be analyzed, and new optimal time settings will be applied in future. A robust technique for detecting movement period could be explored. And formal real-time experiments could be conducted such as a motion test to analyze the accuracy of different algorithms and different sensor configurations to verify the real-time performance of the proposed multi-sensor fusion model on stroke patients. In addition, it may be necessary to consider reducing the number of gestures to improve the accuracy of recognition and performance of the system in future research and commercial product development.

A limitation of this proposed study is that we only included hand gestures and did not include arm reaching movements. To prevent the negative effect of arm movement variation on physiological information-based hand gesture recognition (Fougner et al., 2011), users were asked to drop their hands naturally on both sides of the body to perform the corresponding hand gestures, which didn’t restore the ADLs completely. To make the system more practical, the experiment could be carried out in a semi-natural or total-natural environment in which patients could perform natural ADLs-related movements, such as drinking water, and a more practical model could be developed. Future work could also focus on increasing the robustness of the proposed system, addressing the problem of decreased recognition accuracy caused by sensor picking. The effectiveness of this approach in improving patient upper limb motor function and cognitive function would require testing over an extended period of time, ideally through a long-term, randomized controlled trial. The questionnaire results in this were subjective preliminary indicators that the proposed system could be acceptable for future long-term testing. Electromyography sensors were located 10 cm away from the elbow in this study and not normalized for participant limb length. Questionnaires in future studies should also include standard questionnaires such as System Usability Scale (Kortum et al., 2008) or User Experience Questionnaire (Laugwitz et al., 2008).

The proposed approach validates the advantages of multi-sensor fusion and its application prospects in the field of rehabilitation. Motion pattern recognition plays an important role in both exoskeleton-based rehabilitation training systems and daily life assistance systems. The multi-sensor fusion model we proposed has the potential to be applied on active assisted robotic rehabilitation systems or active ADLs-assisted orthoses to improve their motion recognition performance on the affected side. For the wider application of this approach, a low-cost system will be developed and verified in future research.

## Conclusion

This study proposes a wearable serious-game-based training approach for the rehabilitation of both upper limb motor function and cognitive function. A multi-sensor fusion model was developed for the movement recognition of stroke patients with upper limb dysfunction. Two movement classification-based serious games were developed to train patients’ attention and memory. An experiment involving stroke patients with different levels of upper limb impairments was performed to validate the effectiveness of the proposed approach. Results showed that the sensing and algorithm configurations used in the proposed rehabilitation approach could classify a variety of ADLs related to fine movements. The proposed serious game approach stimulated patients’ enthusiasm for rehabilitation and guided them to actively perform repeated movements. The proposed training approach has the potential to be used in both clinical-based and home-based environments by stroke patients to improve upper extremity motor function and cognitive function. The multi-sensor fusion method can improve the motion recognition performance of stroke patients. This effective model can be used both in unassisted serious-game-training systems and also in the active robotic-assisted rehabilitation system or ADLs-based orthosis.

## Data Availability

The raw data supporting the conclusions of this article will be made available by the authors, without undue reservation.

## Abbreviations

ADLs, activities-of-daily living; CG, cylinder grip; DT, decision tree; FMA, Fugl Meyer Assessment; FMG, force myography; FMUE, Fugl Meyer Upper Extremity; FP, forearm pronation; FS, forearm supination; HG, hook-like grasp; IMU, inertial measurement units; KNN, k-nearest neighbor; LDA, linear discriminant analysis; ME, mass extension; MF, mass flexion; NM, no-motion; O, opposition; PCC, Pearson correlation coefficient; RF, random forest; sEMG, surface electromyography; SG, spherical grip; SVM, support vector machine; TA, thumb adduction; WE, wrist dorsiflexion; WF, wrist volar flexion; WMFT, Wolf Motor Function Test.
