# Supplementary material for: Proposal of a Wearable Multimodal Sensing-Based Serious Games Approach for Hand Movement Training After Stroke
Source: Front Physiol. 2022 Jun 3;13:811950. doi: 10.3389/fphys.2022.811950 (PMC9204487; doi:10.3389/fphys.2022.811950)
Supplement: Supplementary file 1 [file DataSheet1.PDF]

Supplementary Material

1 Patient inclusion criteria

The inclusion criteria of this experiment were: aged 18 to 80 with upper extremity dysfunction after stroke; mini-mental state examination (MMSE) score greater than 24; the modified Ashworth scale for upper extremity spasticity less than 3; and a Brunnstrom stage for hand greater than 1. Patients with pain in the upper extremity, neglect, and aphasia were excluded.

2 Interface of the instructional software

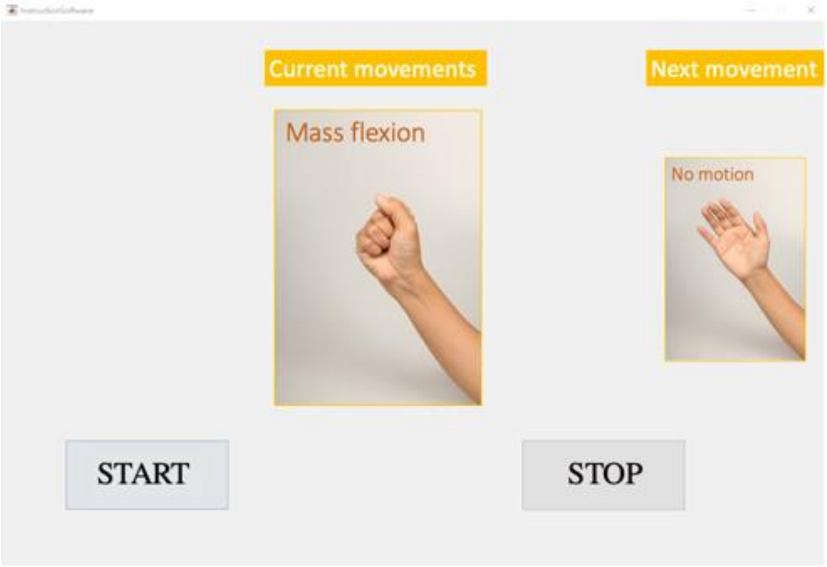

Supplementary Figure 1. Interface of the instruction software. The start button was used to start each trial of the test, and then the software would automatically time the current movement and change to the next movement. The stop button was used to stop the system if some testing mistake occurred.

3 Grouping of the different movements

SUPPLEMENTARY TABLE 1
MOVEMENT GROUPING IN SERIOUS GAMES

**Find the Sheep**	**Group 1**	Mass flexion	Opposition	Wrist dorsiflexion
	**Group 2**	Mass extension	Cylinder grip	Wrist volar flexion
	**Group 3**	Thumb adduction	Spherical grip	Forearm pronation
	**Group 4**	Hook-like grasp	No motion	Forearm supination
**Best Salesman**	**Group 1**	Thumb adduction	Spherical grip	Forearm pronation
	**Group 2**	Cylinder grip	Forearm pronation	Forearm supination
	**Group 3**	Hook-like grasp	Opposition	Forearm supination

4 Feature formulas

$\boldsymbol{MAV}$:

$$\bar{\mathbf{x}}_i = \frac{1}{N}\sum_{i=1}^{N}|x_i|$$

$\boldsymbol{WL}$:

$$l_k = \sum_{i=1}^{N-1}|x_{i+1} - x_i|$$

$\boldsymbol{ZC}$:

$$x_i x_{i+1} < 0$$

$$\textit{and}$$

$$|x_{i+1} - x_i| \geq \epsilon$$

$$SSC: \quad (x_{i+1} - x_i)(x_i - x_{i-1}) < 0$$

$$and$$

- $$|x_i - x_{i+1}| \geq \epsilon \ or \ |x_i - x_{i-1}| \geq \epsilon$$

$$AR: \quad x_k = -\sum_{i=1}^{N} a_i x_{k-i} + e_k$$

5 Different cutoffs - Statistical analysis

The starting cutoff for training data, the starting cutoff for test data, and the amount of voting samples. The ranges of the training data were set up from the 0th to the 5.5th second, from the 1st to the 5.5th second and from the 2nd to the 5.5th second. The test data could be started from the 0th, the 1st, and the 2nd second, and the length of voting samples could be 10 or 20. To find the optimal cutoff settings, the accuracies of 12 hand gestures classification under different combinations of cutoffs were calculated.

Real-time classification after grouping under optimal cutoff setting was simulated to validate the performance of the real-time-based serious games. The simulated hand gesture classification accuracies of patients playing each serious game under best cutoffs were calculated and compared to the accuracy of the cutoff we used. The statistical analysis was set to $p<0.05$.

6 Different cutoffs - Results

The accuracies of the simulated real-time classification with different training and test data ranges are shown in Supplementary TABLE 2. The test time started from the 2nd second had better performance than the test time started from the 1st second and the 0th second. In addition, the results predicted and voted by 20 samples were better than the results predicted and voted by 10 samples in the same circumstances.

Comprehensively considering the accuracy of recognition and the practicality of online recognition, two ideal combinations were selected, and real-time classification after grouping was simulated to validate the performance of the real-time-based serious games. One of the best setups for real-time classification was the training data ranged from the 0th to the 5.5th second, the test data started from the 1st second, and the length of voting set at 20. In this circumstance, the classification accuracy of each group (Supplementary TABLE 1) ranged from 86.7% to 94.7%, with the average classification accuracies of the two serious games being 90.5% and 92.2%, respectively. The other setup we selected for real-time classification had the training data ranging from the 2nd to the 5.5th second, the test data starts from the 2nd second, and the length of voting is 10. With these settings, the classification accuracy of each group (Supplementary TABLE 1) ranged from 90.7% to 94%, with the average classification accuracies of the two serious games being 91.8% and 92.4%, respectively. The results of the three cases (the two mentioned above and the one we actually used) are shown in Supplementary Figure 2. Compared to the settings we used, there are significant improvements in the real-time classification

accuracies when using the settings mentioned above.

SUPPLEMENTARY TABLE 2
THE SIMULATED REAL-TIME CLASSIFICATION ACCURACIES OF DIFFERENT COMBINATIONS OF CUTOFFS OF TRAINING DATA AND TESTING DATA FOR 12 MOVEMENTS

		**Training Data (Start-end)**		
		**0-5.5**	**1-5.5**	**2-5.5**
**Testing Data (Start [vote length])**	**0 [10]**	0.45	0.37	0.35
	**0 [20]**	0.61	0.53	0.49
	**1 [10]**	0.79	0.75	**0.70**[a]
	**1 [20]**	**0.83**[b]	0.81	0.77
	**2 [10]**	0.84	0.84	**0.83**[c]
	**2 [20]**	0.85	0.84	0.84

The unit of start and end time is second, and the unit of vote length is sample. For example, 1[10] means the first ten samples starting from the 1st second testing data were used to vote the final predicted movement. 2-5.5 means the training data cutoff is from the 2nd to the 5.5th second. [a] is the classification accuracy when using the cutoffs we applied. [b] is the classification accuracy when using the cutoff 1 we selected, and [c] is the classification accuracy when using the cutoff 2 we selected.

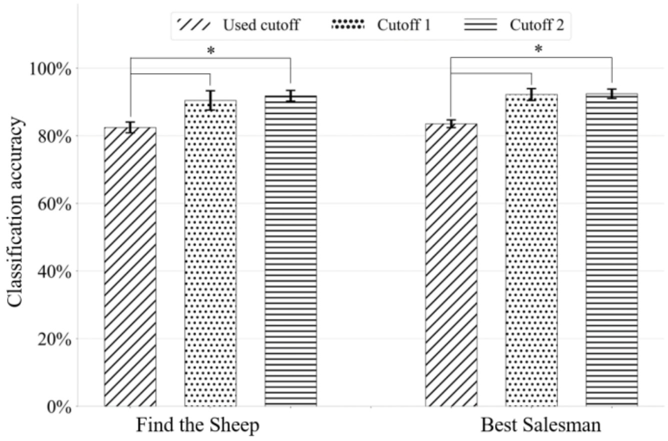

Supplementary Figure 2. The simulated real-time classification accuracy while playing each game when applying different cutoffs. Used cutoff, Cutoff 1 and Cutoff 2 correspond to cutoffs a, b and c in Supplementary TABLE 2. * represents statistical significance ($p<0.05$). Compared to applying the Used cutoff, the accuracies of applying Cutoff 1 and Cutoff 2 were significantly improved.
